# FireProt^ASR^ 2.0: evolution-guided Design of Protein Ancestors and Successors with phylogenetics and machine learning

**DOI:** 10.1093/bib/bbag436

**Published:** 2026-08-27

**Authors:** Pavel Kohout, David Lacko, Milos Musil, Simeon Borko, Martin Stepanek, Jan Velecky, Petr Kabourek, Rayyan Tariq Khan, Monika Rosinska, Jiri Damborsky, Stanislav Mazurenko, David Bednar

**Affiliations:** Loschmidt Laboratories, Department of Experimental Biology and RECETOX, Faculty of Science, Masaryk University, Kamenice 753/5, Brno 625 00, Czech Republic; International Clinical Research Centre, St. Anne’s Hospital, Pekarska 53, Brno 656 91, Czech Republic; Loschmidt Laboratories, Department of Experimental Biology and RECETOX, Faculty of Science, Masaryk University, Kamenice 753/5, Brno 625 00, Czech Republic; International Clinical Research Centre, St. Anne’s Hospital, Pekarska 53, Brno 656 91, Czech Republic; Loschmidt Laboratories, Department of Experimental Biology and RECETOX, Faculty of Science, Masaryk University, Kamenice 753/5, Brno 625 00, Czech Republic; International Clinical Research Centre, St. Anne’s Hospital, Pekarska 53, Brno 656 91, Czech Republic; Department of Information Systems, Faculty of Information Technology, Brno University of Technology, Bozetechova 2, Brno 612 00, Czech Republic; Loschmidt Laboratories, Department of Experimental Biology and RECETOX, Faculty of Science, Masaryk University, Kamenice 753/5, Brno 625 00, Czech Republic; International Clinical Research Centre, St. Anne’s Hospital, Pekarska 53, Brno 656 91, Czech Republic; Loschmidt Laboratories, Department of Experimental Biology and RECETOX, Faculty of Science, Masaryk University, Kamenice 753/5, Brno 625 00, Czech Republic; Department of Information Systems, Faculty of Information Technology, Brno University of Technology, Bozetechova 2, Brno 612 00, Czech Republic; Loschmidt Laboratories, Department of Experimental Biology and RECETOX, Faculty of Science, Masaryk University, Kamenice 753/5, Brno 625 00, Czech Republic; International Clinical Research Centre, St. Anne’s Hospital, Pekarska 53, Brno 656 91, Czech Republic; Loschmidt Laboratories, Department of Experimental Biology and RECETOX, Faculty of Science, Masaryk University, Kamenice 753/5, Brno 625 00, Czech Republic; International Clinical Research Centre, St. Anne’s Hospital, Pekarska 53, Brno 656 91, Czech Republic; Loschmidt Laboratories, Department of Experimental Biology and RECETOX, Faculty of Science, Masaryk University, Kamenice 753/5, Brno 625 00, Czech Republic; International Clinical Research Centre, St. Anne’s Hospital, Pekarska 53, Brno 656 91, Czech Republic; Loschmidt Laboratories, Department of Experimental Biology and RECETOX, Faculty of Science, Masaryk University, Kamenice 753/5, Brno 625 00, Czech Republic; International Clinical Research Centre, St. Anne’s Hospital, Pekarska 53, Brno 656 91, Czech Republic; Department of Information Systems, Faculty of Information Technology, Brno University of Technology, Bozetechova 2, Brno 612 00, Czech Republic; Loschmidt Laboratories, Department of Experimental Biology and RECETOX, Faculty of Science, Masaryk University, Kamenice 753/5, Brno 625 00, Czech Republic; International Clinical Research Centre, St. Anne’s Hospital, Pekarska 53, Brno 656 91, Czech Republic; Loschmidt Laboratories, Department of Experimental Biology and RECETOX, Faculty of Science, Masaryk University, Kamenice 753/5, Brno 625 00, Czech Republic; International Clinical Research Centre, St. Anne’s Hospital, Pekarska 53, Brno 656 91, Czech Republic; Loschmidt Laboratories, Department of Experimental Biology and RECETOX, Faculty of Science, Masaryk University, Kamenice 753/5, Brno 625 00, Czech Republic; International Clinical Research Centre, St. Anne’s Hospital, Pekarska 53, Brno 656 91, Czech Republic

**Keywords:** ancestral sequence reconstruction, successor sequence prediction, variational autoencoders, evolutionary modeling, machine learning, protein design

## Abstract

Evolution-guided protein design remains one of the most effective strategies for engineering proteins with enhanced stability, activity, or specificity. To make these approaches more accessible, we previously developed FireProt^ASR^—a fully automated pipeline for ancestral sequence reconstruction (ASR). Here, we present FireProt^ASR^ 2.0, a significantly enhanced version that extends the design space beyond ancestral inference by integrating a successor sequence predictor (SSP) and a generative model based on variational autoencoders (VAEs). These new modules enable both ‘prospective’ and ‘retrospective’ evolutionary design strategies. The SSP module predicts likely future mutations based on site-wise evolutionary trends, and the method was previously validated through *in silico* benchmarks, demonstrating improvements in thermostability and activity. The VAE module captures global evolutionary constraints in a low-dimensional latent space, from which novel functional ancestral-like variants can be sampled. The VAE-based design strategy was previously validated experimentally on the haloalkane dehalogenase family, yielding variants with enhanced thermostability while maintaining catalytic activity. Both these modules are newly available in FireProt^ASR^ in a fully automated pipeline, guiding the users via an interactive graphical user interface. With expanded functionality, modernized user interface, and a more robust backend, FireProt^ASR^ 2.0 provides a comprehensive, accessible, and fully automated platform for evolutionary-based protein engineering (https://loschmidt.chemi.muni.cz/fireprotasr/).

## Introduction

The vast sequence space of proteins presents a significant challenge in protein design, as only a tiny fraction of possible sequences are functional [1]. Evolutionary principles help navigate this immense space by focusing on protein family-related sites and mutations, effectively constraining design efforts to functionally relevant regions [2–4]. This targeted approach enhances the efficiency of mutagenesis compared to non-evolutionary-based methods, increasing the success rate of computational designs while reducing the labour and material costs associated with extensive experimental work [4–6].

A well-established and powerful computational method for evolutionary-based protein design is ancestral sequence reconstruction (ASR) [7]. ASR leverages homologous sequences aligned in a multiple sequence alignment (MSA) and an inferred phylogenetic tree to reconstruct ancestral nodes. This method has been successfully applied to generate functional, promiscuous, substrate-shifted, and thermostabilized proteins, which can be further optimized for specific applications [8, 9]. For instance, ASR has been used to create Combinatorial Libraries of Ancestors for Directed Evolution (CLADE), which enables the identification of stabilizing mutations in cytochrome P450 enzymes [10, 11].

Despite its advantages, ASR remains a labour-intensive process requiring multiple manual steps, including MSA generation, phylogenetic tree inference, ancestral sequence prediction, and subsequent sequence refinement, particularly for gap correction. To address these challenges, we previously developed FireProt^ASR^ [12], a fully automated web tool streamlining all ASR steps. The FireProt^ASR^ workflow enables the reconstruction of ancestral enzymes related to a user-provided sequence and catalytic residues using the EnzymeMiner tool and an intuitive, user-friendly interface. Furthermore, FireProt^ASR^ allows interaction with the pipeline at multiple levels, enabling users to incorporate their prior knowledge into the reconstruction process. FireProt^ASR^ has been experimentally validated and has served as a valuable resource in multiple studies, significantly improving the accessibility of ASR-based protein design [12–19]. Notably, the full power of FireProt^ASR^ was examined in an exhaustive study of *ene reductases* [13]*.* In this study, the complete phylogenetic tree of 57 closely related wild-type ene reductases and 56 counterpart ancestral enzymes was characterized, and statistically compared their biocatalytic properties displaying enhanced average thermostability (by 9 °C) compared to their wild-type counterparts. Building on the previous work of Livada et al. [13] and exploring the uncertainties of ASR, the smart CLADE library was created. When combined with machine learning (ML), it led to the identification of additional active and thermostable variants [14].

Notwithstanding the successful use cases of FireProt^ASR^, the current version has two main limitations. First, FireProt^ASR^ does not allow investigating evolutionary trends in the generated phylogenetic trees. However, the ability to predict the next step in evolution could open new opportunities for targeted protein optimization, particularly for improving activity, which is one of the desired industrial objectives, hidden in complex evolutionary processes [20, 21]. One approach to achieving this is by identifying evolutionary trends in selected physicochemical feature spaces of amino acids, such as those represented by AAIndex values, using regression models. We have recently published a method, Successor Sequence Predictor (SSP), that allows extracting and extending observable evolutionary trends from phylogenetic trees to leverage their signals to predict the future trajectory of a protein’s adaptation [22]. The mutations suggested by this method were shown to further enhance the evolutionary optimization of specific properties across four in silico benchmarks, each reflecting a distinct design objective: solubility, thermostability, selectivity, and activity. Notably, the method exhibited particularly promising results in improving enzymatic activity, as demonstrated on a deep mutational scanning dataset of aminoglycoside 3′-phosphotransferase [23].

The second limitation is the lack of data-driven methodologies that extend beyond conventional phylogenetic inference. In recent years, ML methods have gained significant attention, offering promising avenues for capturing the complex features of protein evolutionary landscapes [24–27]. Of particular interest are generative models, which have shown remarkable success in protein engineering [28–30]. These protein language models, trained on large datasets comprising tens of thousands of sequences, can generate novel protein variants or suggest new mutations (zero-shot) [31]. While they often incorporate MSA data, they do not explicitly account for evolutionary trends. Our recently published VAE-based pipeline addressed this gap by modeling a continuous evolutionary trajectory in latent space, yielding proteins with up to 9 °C higher melting temperatures (average + 3 °C) and up to 3.5-fold increased activity [25].

In this work, we present a major update to the original FireProt^ASR^, designed to address the evolving needs in protein evolution prediction and integrate the latest advancements in machine learning. As outlined above, the update introduces two novel methods: SSP and evolutionary structured latent spaces using variational autoencoders, now fully implemented in a seamless automated workflow. Additionally, we have revamped the user interface to incorporate current best practices in web tool development, ensuring long-term tool maintenance and smooth operation, even on older hardware. These updates significantly expand the applicability and usability of FireProt^ASR^ 2.0.

## Results

### Workflow: Novel features

**Figure 1:**
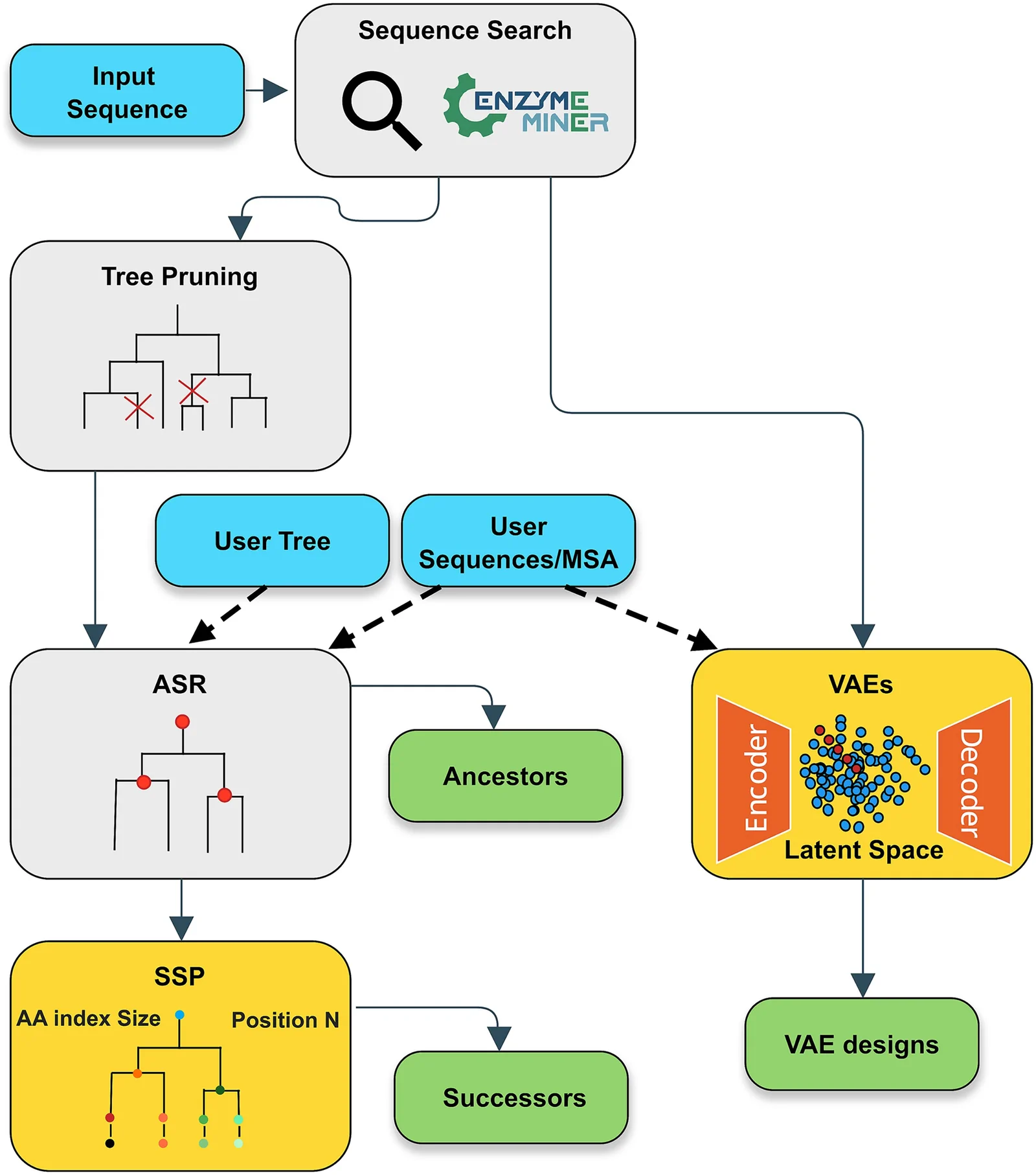
Scheme of FireProt^ASR^ computational workflow. New methods are the variational autoencoders (VAEs) and the sequence successor predictor (SSP) (in yellow). The user can provide input in the form of a single sequence, a set of user-defined sequences, or an multiple sequence alignment (MSA), and optionally a phylogenetic tree. Input sequences are aligned and processed by the ancestral sequence reconstruction (ASR) or VAE module. A custom MSA can be supplied to incorporate prior knowledge into the analysis. Additionally, if an MSA is provided, the ASR module can be guided by a user-defined phylogenetic tree, which will be respected during ancestral sequence reconstruction.

The basic workflow of the FireProt^ASR^ 2.0 method is outlined in Fig. 1. This fully automated approach infers evolutionarily related variants of an extant sequence using two independent computational branches, each incorporating novel computational analyses. The process begins with the collection of biologically relevant homologous sequences from genomic databases, which are subsequently aligned into a multiple-sequence alignment. Further processing of the homologous sequences is implemented depending on the selected computational analysis.

To improve robustness when processing large mining results, FireProtASR 2.0 introduces an additional diversity-preserving subsampling procedure. When the number of mined sequences exceeds the available computational resources. As part of the reduction step, the sequence set is dynamically reduced using clustering by progressively decreasing the sequence identity threshold in 5% increments while preserving sequence diversity, until the number of sequences falls within computationally manageable limits. This preprocessing step prevents failures during downstream analyses and enables stable processing of large sequence datasets.

As in the previous version, when ASR is utilized, the collected, homologous sequences are first reduced to a representative subset using state-of-the-art methods (Phase 1). This reduced set is then used to infer ancestral nodes in a phylogenetic tree. A new feature in FireProt^ASR^ 2.0 extends this phylogenetic information by generating evolutionary descendants for all extant sequences within the tree (see ‘Sequence Successor Predictor’ below).

Alternatively, the complete set of homologous sequences can be used as input for a novel machine learning-based computational branch that constructs evolutionary latent spaces. The trained machine learning model generates an evolutionarily informed low-dimensional representation, which is then leveraged to design novel protein sequences with evolutionary prior (see ‘Evolutionary structured latent spaces of Variational Autoencoders’ below). An overview of the added features in the new version is highlighted in Table S1.

### Sequence successor predictor module

SSP is a method designed to mimic evolutionary processes by predicting descendant sequences based on observed linear trends in biochemical properties. These trends are captured using a carefully selected set of amino acid indices. Furthermore, SSP relies on evolutionary data in the form of an MSA and a corresponding phylogenetic tree with reconstructed ancestral sequences. These evolutionary datasets are generated in a fully automated manner using the previous version of FireProt^ASR^.

As in the original FireProt^ASR^ pipeline, the user submits a single query sequence, and the system performs a homology search, taking essential residues into account if provided. This ensures that functionally important positions are preserved throughout the evolutionary analysis. The initial set of homolog sequences obtained from the EnzymeMiner search is subjected to several steps of sequence filtration that reduce this dataset to a more manageable size (Fig. 1, Sequence reduction). First, the dataset is refined based on the homolog’s length and identity compared to the query and clustered to a 90% similarity to assure the diversity of the reduced set. The initial phylogenetic tree is constructed using the fast neighbour-joining algorithm and subsequently trimmed by iteratively removing closely related branches to obtain a final set of 150 diverse, yet biologically relevant homologous sequences. Next, the phylogenetic tree is reconstructed employing the more robust maximum-likelihood algorithm and rooted using the minimal ancestral deviation method (Fig. 1, ASR). Finally, the posterior probabilities for each of the standard amino acids in each position of every ancestral node are calculated, and the most likely amino acids are assigned to the individual ancestral sequences. The ancestral sequences are then corrected by replacing amino acids at positions with high indel probability with ancestral gaps [12].

The calculated tree with inferred ancestral sequences serves as the input to the SSP (Fig. 1). In comparison to the original SSP, which generated predictions from multiple alternative phylogenetic trees to account for uncertainty in tree reconstruction, we simplified and fully automated the workflow to operate on a single phylogenetic tree instead of multiple trees. This modification significantly reduces computational demands, from days to hours, while remaining fully compatible with the FireProt^ASR^ pipeline and producing a conservative subset of the original SSP predictions. Although using a single tree reduces explicit sampling of phylogenetic uncertainty, our analyses indicate that the resulting predictions retain the diversity of the original approach while representing a conservative subset of its prediction space (Fig. S2). In addition, the updated workflow applies a streamlined process to all extant sequences, MSA positions, and a selected set of nine amino acid indices (AAindex). These indices, namely molecular weight (FASG760101), melting point (FASG760102), residue volume (GOLD730102), hydrophobicity (WOLR790101), flexibility (BHAR880101), free energy (BULH740101), electrical effect (FAUJ880108), polarity (ZIMJ680103), and isoelectric point (ZIMJ680104), were manually selected to reflect a variety of important physiochemical descriptors [22].

Briefly, the SSP protocol proceeds as follows. First, it extracts ancestral nodes between each extant sequence and the root from the final alignment of all extant and ancestral sequences. At each position, if more than 50% of the values in a column are gaps or the processed position is a gap itself, no prediction is made, and the original amino acid (or a gap) from the extant sequence is retained. As a result, the algorithm does not predict insertions. Additionally, predictions are limited to standard amino acid symbols, so deletions are not supported either. At each position, the same consecutive amino acids along the evolutionary trajectory are grouped, and the number of transitions between different groups is counted. A prediction is made only if there are more than three transitions, ensuring that predictions are limited to positions with a strong evolutionary signal. For such positions, each amino acid along the evolutionary trajectory is represented by its value in a selected AAindex, generating a series of physicochemical feature values. These values are weighted by evolutionary distance from the root and used to train a linear model that predicts the next amino acid, thereby simulating laboratory-driven protein evolution. Finally, based on the extrapolated AAindex feature values, the closest matching amino acid for each AAindex in this multidimensional space is returned as the prediction.

To capture more information about trends in feature values, the ‘*sequentiality’* and *‘trend break’* descriptors are calculated for individual trajectories over individual amino acid indices, capturing key properties of each trajectory. The *trend break* flags the last transition that deviates from the overall direction of change, while *sequentiality* scores the consistency of the trend on a scale from 0 to 100 (see Fig. S1). A perfect score (100) indicates that each step in a positive trend reflects a strictly increasing/decreasing feature value compared to the previous one. These scores are then used to rank successor amino acid predictions for each site and index. The top-ranked predictions are those that exhibit high *sequentiality* and no *break trend* at the penultimate amino acid position within the trajectory. If a *break trend* occurs at the given MSA position and AAindex, the prediction is reverted to the original amino acid. When all predictions produce the same mutation at a given site, these are merged into a single *Mutation-Agreeing Prediction* (MAP). MAP prediction is introduced if there are two or more AAindices agreeing in prediction. In contrast, when any of the AAindices disagree in mutation prediction at a given site, they are categorized as *Site-Agreeing Predictions* (SAPs). Final SAP is calculated only if there are three or more predictions of mutation. Consequently, the best prediction is determined as the compromise of scores given by *sequentiality,* the confidence of the prediction (error between the true AAindex value of the amino acid and the extrapolated value), and its frequency within SAPs. The illustration of successor prediction is shown in Fig. S1.

### Variational autoencoders-based design module

With advancements in machine learning, new approaches have emerged that offer complementary ways to study evolution in ASR. These methods move beyond traditional tree-based inference and allow for a more flexible exploration of sequence space and open novel opportunities for protein engineering. One such approach is VAEs, a type of deep generative learning models whose goal is to learn the input data distribution. VAEs consist of an encoder that maps input to a structured latent space and a decoder that reconstructs sequences from sampled latent points. The latent space is modeled as a probability distribution, controlled by mean and variance parameters, ensuring smooth interpolation between related sequences. Training a VAE involves optimizing a loss function that balances accurate sequence reconstruction and latent space regularization using Kullback–Leibler (KL) divergence.

When applied to MSA, VAEs structure the latent space in a way that naturally reflects evolutionary relationships as ancestral sequences are positioned closer to the latent space origin than their extant counterparts [32]. This observation can be leveraged to generate machine learning-based ancestral sequences by guiding sequence sampling along the evolutionary trajectory embedded within the latent space. To achieve this, we developed the straight-line evolutionary strategy, systematically exploring the latent space to infer ancestral-like sequences (Fig. 1, VAEs).

The process begins with selecting a query sequence and refining the MSA to enhance the evolutionary signal while reducing noise from query-distinct proteins. This preprocessing includes filtering out MSA columns that lack the query amino acid or have less than 80% occupancy by other sequences, removing sequences shorter than 40% of the processed MSA width, and eliminating those with less than 50% overlap with the query. The preprocessed MSA is randomly split into training (90%) and validation (10%) sets, ensuring that the query sequence is included in the training set. Model training is performed using early stopping, with the Adam optimizer set to a learning rate of 0.001. Training is halted if the loss function does not improve for three consecutive rounds. Once training is complete, the query sequence is encoded into its latent representation, and a straight path is drawn from this point toward the latent space origin, mimicking the evolutionary trajectory of ancestral sequences. The path is divided into equal intervals, and sequences at these positions are reconstructed by the decoder.

Several key statistical parameters are calculated to assess the designed sequences, including average reconstruction probability (probability), sequence similarity to the query protein, similarity to the closest sequence from the preprocessed MSA, and the number of insertions/deletions. Average reconstruction accuracy is measured by sampling 500 sequences from the latent space (using the encoder’s mean and variance) and calculating their average identity to the original input sequence. The results are visualized to identify variants with promising statistical properties for further analysis.

### Webserver inputs

**Figure 2:**
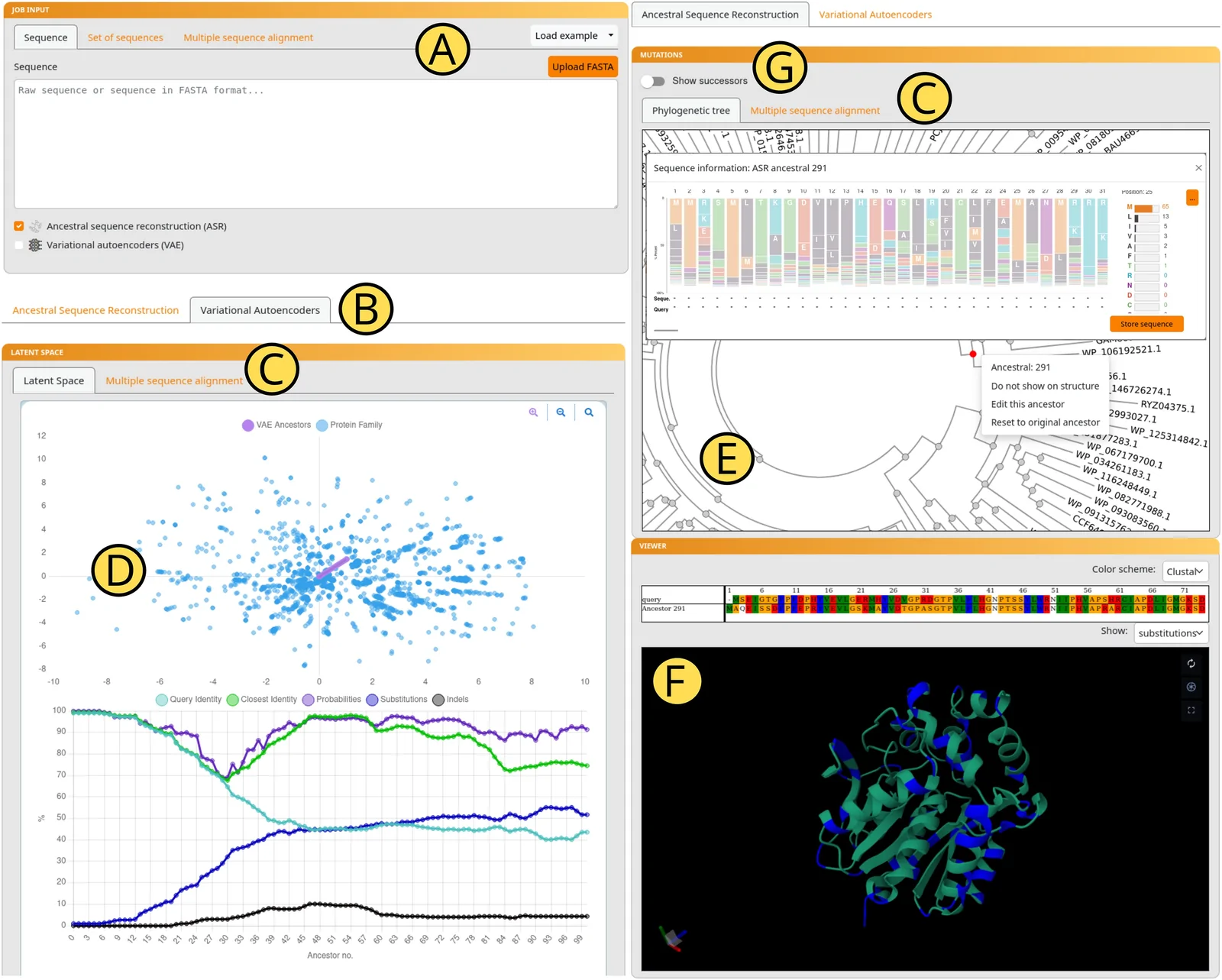
FireProt^ASR^ graphical interface showing multi-data type input form and results for haloalkane dehalogenase DhaA (UniProt ID P0A3G2). (**A**) The input form accepts a single sequence, a set of sequences, or an MSA. Users can then choose the computational method to be applied. (**B**) The result page includes tabs for method-specific visualizations based on the selected input. (**C**) Separate result views are provided for VAE and ASR + SPP calculations, each with a dedicated tab for MSA visualization. (**D**) VAE result visualization. Top: Latent space of mined sequences (blue), highlighting reduced ASR sequences (dark purple) and the sampled evolutionary trajectory (light purple). Bottom: Statistical profiles for generated sequences. (**E**) ASR results are displayed alongside the phylogenetic tree, with an amino acid posterior probability window for customizing generated ancestors (available for both VAE- and ASR-derived sequences). (**F**) The query structure with mapped substitutions from the selected ancestor. (**G**) Visualization and analysis of predicted successor sequences for all extended nodes in the phylogenetic tree. Please refer to https://loschmidt.chemi.muni.cz/fireprotasr/example for a step-by-step example.

FireProt^ASR^ 2.0 allows users to initiate the workflow from various stages depending on the input provided (Fig. 1). At a minimum, a query protein sequence in plain text or FASTA format is required. Users may also choose to supply a precompiled set of homologous sequences in FASTA format or an MSA. Additionally, both rooted and unrooted phylogenetic trees can be uploaded in Newick format to further customize the reconstruction process (Fig. 2A).

The analysis path depends on the user’s input. Uploading an MSA or a FASTA file with up to 150 sequences enables ASR, while the VAE method requires larger datasets and is available only with 150 or more sequences. If a phylogenetic tree is provided, only ASR is available. When starting from a single sequence, users can run ASR, VAE, or both. In this case, only a table of essential residues, automatically identified from SwissProt [33], is shown, but can be manually edited to include sequences with relevant catalytic activity. By default, tools run with pre-set parameters.

Advanced mode allows users to customize sequence filtering thresholds, VAE settings for filtering, training, and sampling; and selection of input sequences, especially when prior knowledge is available (see ‘Selection and reduction’ in [12]). The VAE filtering settings allow users to reduce the dataset to 90% sequence identity and to retain specific sequences regardless of the filtering criteria defined earlier. In the training settings, users can adjust the number of training *epochs* (with early stopping applied by default), the number of neurons in the *dense layer* (default is the length of the query sequence), the *batch size* (default is the number of input sequences), and the initial weight decay (WD) value and its dynamic linear decrease to 0.0 during the first 1000 epochs. Adjusting the batch size and modifying or disabling weight decay (WD) can help the model better focus on reconstruction. In contrast, high WD values without dynamic decay can negatively affect sequence reconstruction accuracy, creating ancestors with lower sequence similarity to the query right from the start of the latent evolutionary trajectory, but increase the representational potential of created latent spaces (see Variant Selection section). In the VAE reconstruction settings, users can adjust the number of reconstructed ancestors generated using the straight-line evolutionary strategy, effectively controlling the resolution of evolutionary changes over time in the latent space (default is 100). Also, the starting point for the straight-evolutionary strategy can be changed via *the evolutionary query* field (default is the query).

### Webserver outputs

Progress can be monitored through the *calculation browser*. Once complete, results can be downloaded as a ZIP archive or explored further via the *Result page*. This page is divided into interactive panels for individual computational branches that support the visualization and design of ancestral enzymes (Fig. 2B). Within each panel, results are further separated into tabs displaying the computational outputs and the final MSA used as input for the respective method (Fig. 2C).

For VAE-based results (Fig. 2D), two primary visualizations are provided: (i) the latent space of the input MSA and (ii) the statistical profiles of the sampled ML-ancestral sequences generated along a straight evolutionary trajectory from the Evo-Query sequence (query by default). The latent space plot illustrates sequence diversity and its coverage by sequences selected for phylogenetic reconstruction in the ASR branch. It also indicates positions of the points sampled along the straight-line evolutionary path and decoded into VAE-based ancestral sequences. The latent space is interactive, allowing users to select and examine details of individual ancestors and edit them (Fig. 2E, drop menu). This interaction is bidirectional; selecting a point in the latent space highlights the corresponding statistical profile below, and vice versa. Additionally, mutations between the selected ancestor and the evolutionary query can be visualized on the protein structure (Fig. 2F).

The common ASR and SSP results page allows users to interact with a phylogenetic tree constructed from a reduced set of sequences, with inferred ancestral sequences displayed at the corresponding internal nodes (Fig. 2E). By selecting an ancestral node, users can examine the posterior distribution of amino acid probabilities, similar to the VAE-based ancestor view, and optionally modify the predicted residues through an interactive modal window. This interface also offers an automated option to revert insertions and deletions back to the query sequence, effectively restricting modifications to substitutions only. The distribution of mutations, including insertions and deletions, can be visualized on the query structure (Fig. 2F, blue), with visibility controlled via the ‘Show’ drop-down menu. Additionally, users can switch the ASR view to display predicted successors instead of the extant proteins on the leaves of the evolutionary tree (Fig. 2G), enabling further exploration of successor sequences and their structural mapping onto the query protein.

### Validation

The original ASR pipeline has been previously thoroughly validated through experimental studies, demonstrating its ability to produce stabilized and functionally active protein designs [12–19]. Here, we revalidated the newly implemented ASR module by reproducing the original results for haloalkane dehalogenase DhaA [12]*.* The two new modules also implement thoroughly tested methods. Briefly, SSP was evaluated in silico using four benchmark datasets assessing the performance on homolog sets of levoglucosan kinase (solubility), cold shock protein CspB (thermostability), ADP-ribosylarginine hydrolase (selectivity), and aminoglycoside 3′-phosphotransferase (activity) [22]. The VAE pipeline was experimentally validated on the protein family of haloalkane halogenases, producing stabilized (up to +9 °C) and active designs with a 3.5-fold increase in activity compared to the template [25]. The overall pipeline settings were tested on four selected systems (DhaA, LinB, CSP, and SAK).

### Variant selection

Computational modules such as ASR, SSP, or VAE typically generate a large pool of candidate variants, from which users must select the most promising ones for wet-lab validation. Selection strategies depend on the intended goal: (i) designing a specific protein or (ii) exploring the sequence space.

In the first scenario, candidate selection in the ASR or VAE module typically follows a shared strategy: navigating between the query sequence and the root of the phylogenetic tree or the origin of the latent space. Choosing nodes close to the query tends to be safer but may yield limited gains in protein properties, while exploring more distant ancestors can be riskier yet potentially more rewarding. In general, ASR has proven to be a robust design strategy [13, 34]. A commonly used approach within our team involves selecting several nodes located on the query-to-root path in the phylogenetic tree (nodes close to the query, midpoints, and close to the root). Alternatively, users may prioritize ancestral nodes that represent common ancestors of both the query and another wild-type sequence in the dataset that is characterized and exhibits desirable properties or a distinct latent space representation. By this common ancestor strategy, even dual activity enzymes can be obtained [12].

For VAE-based designs, the safest approach involves selecting ancestral sequences with relatively high similarity to known sequences in the dataset (preferably those with available PDB structures), and with minimal or no insertions or deletions. Careful consideration and rational filtering of insertions and deletions are strongly advised, as these changes can significantly impact the resulting protein structure and function. The user interface also offers the option to revert all insertions and deletions relative to the query sequence using the Sequence Design Model dialogue. This feature can help reduce the risk of introducing biases originating from the broader MSA. In the case of SSP, the method has been benchmarked primarily for single-point mutations. Therefore, applying individual mutations suggested by SSP in a targeted region of the protein is likely the most reliable approach. Note that potential epistatic interactions between mutations have not been evaluated in this framework.

In the second strategy, the exploration of a broader sequence space, users can leverage the latent space in combination with projections of pruned extant variants onto that space. For this purpose, we recommend running the VAE with modified advanced settings: set the weight decay to 0.1 and disable the dynamic decay option. These adjustments promote the learning of general evolutionary patterns across the entire input dataset, rather than focusing on precise reconstruction. Additionally, selecting ‘sequences from ASR’ (see Fig. 2D) from distinct clusters within the latent space can facilitate a more diverse and exploratory search through sequence space.

## Discussion

Evolution-guided protein design has proven to be a highly effective strategy for navigating the vast and largely uncharted protein sequence space [3, 35]. Focusing mutational efforts on evolutionarily constrained regions increases the likelihood of producing functional and stable variants while reducing the need for extensive experimental screening [36–38]. To streamline the application of this strategy, we previously developed FireProt^ASR^, a fully automated pipeline that integrates all essential steps of ASR [12]. FireProt^ASR^ has been experimentally validated and successfully applied in multiple studies [12–19].

While ASR provides valuable insight into historical protein states, evolutionary information can also be leveraged in two new paths. On the one hand, understanding the trajectory of accumulated mutations offers new opportunities for targeted protein optimization by anticipating future adaptations. To extend ASR-based design in this direction, we introduced a SSP, enabling the inference of likely future variants based on evolutionary trends [22]. The current SSP framework focuses on single-residue substitutions and was not explicitly validated for epistatic interactions. Therefore, future extensions could leverage newly emerging large-scale mutational scanning datasets such as FLIP2 [39] and Tran et al. [40], which contain multiple-site mutant protein landscapes suitable for developing and validating epistasis-aware models. This ‘prospective’ component complements ASR by expanding the design space beyond ancestral nodes and guiding mutagenesis with enhanced precision. On the other hand, the rapid advancement of machine learning has opened new, powerful avenues for modeling complex evolutionary relationships in latent spaces [25]. By integrating generative VAE models, users can learn from large sequence datasets and interpretable evolutionary embeddings. VAEs provide a unified framework for protein engineering that is both data-driven and evolution-aware [25, 32]. With the integration of these new modules into an accessible web-based platform, FireProt^ASR^ 2.0 advances evolution-guided protein design, making it broadly available to the scientific community. Its intuitive interface and automated workflows eliminate the need for prior bioinformatics expertise, enabling researchers from diverse backgrounds to harness state-of-the-art computational methods for protein engineering.

**Key Points**

- FireProtASR 2.0 extends ancestral sequence reconstruction with both **prospective** (Successor Sequence Prediction) and **retrospective** (Variational Autoencoders, VAE) evolutionary design strategies based on machine learning.
- The **Successor Sequence Predictor (SSP)** enables prediction of likely future mutations from phylogenetic trends.
- A **VAE-based module** captures evolutionary landscapes in the latent space and enables the generation of machine-learning-based ancestral variants.
- The platform integrates phylogenetics and machine learning into a **fully automated, user-friendly web server**.

## Supplementary Material

Kohout_FPASR2_Briefings_R1_SI_bbag436

## Data Availability

The FireProtASR 2.0 web server is freely available at https://loschmidt.chemi.muni.cz/fireprotasr/. All data used in this study are derived from publicly available databases as cited. Example datasets and results are accessible through the web interface. Source code and additional materials are available from the corresponding authors upon reasonable request.
