# Supplementary material for: FireProtASR 2.0: evolution-guided Design of Protein Ancestors and Successors with phylogenetics and machine learning
Source: Brief Bioinform. 2026 Aug 27;27(4):bbag436. doi: 10.1093/bib/bbag436 (PMC13518065; doi:10.1093/bib/bbag436)
Supplement: Supplementary file 1 [file kohout_fpasr2_briefings_r1_si_bbag436.pdf]

Supplementary Information

FireProt^{ASR} 2.0: Evolution-Guided Design of Protein Ancestors and Successors with Phylogenetics and Machine Learning

AUTHORS

Pavel Kohout[1,2], David Lacko[1,2], Milos Musil[1,2,3], Simeon Borko[1,2], Martin Stepanek[1,3], Jan Velecky[1,2], Petr Kabourek[1,2], Rayyan Tariq Khan[1,2], Monika Rosinska[1,2,3], Jiri Damborsky[1,2], Stanislav Mazurenko[1,2,*], David Bednar[1,2,*]

[1] Loschmidt Laboratories, Department of Experimental Biology and RECETOX, Faculty of Science, Masaryk University, Brno 625 00, Czech Republic
[2] International Clinical Research Centre, St. Anne's Hospital, Brno 656 91, Czech Republic
[3] Department of Information Systems, Faculty of Information Technology, Brno University of Technology, Brno 612 00, Czech Republic

Comparison of FireProt^{ASR} versions

Table S1: Comparison of advances delivered in the new tool FireProt^{ASR} 2.0 to its predecessor FireProt^{ASR} 1.0.

	**Version**		
**Category**	**1.0**	**2.0**	**Effect**
EnzymeMiner 2.0[1] integration	No	Yes	Expanded metagenomic sequence mining
Preprocessing of large mining searches	No	Yes	Robust handling of large sequence datasets through diversity-preserving subsampling
Successor sequence prediction[2]	No	Yes	Prediction of evolutionary successor sequences
Machine learning-based ancestors[3]	No	Yes	VAE-based generation of evolution-guided ancestor-like sequences using large MSA inputs (mined or user-provided)
Machine learning-based ancestor user interface	No	Yes	Interactive 3D structural visualization and annotation of generated ancestors
Successor sequence prediction user interface	No	Yes	Interactive 3D structural visualization of predicted successor sequences

Successor sequence predictor

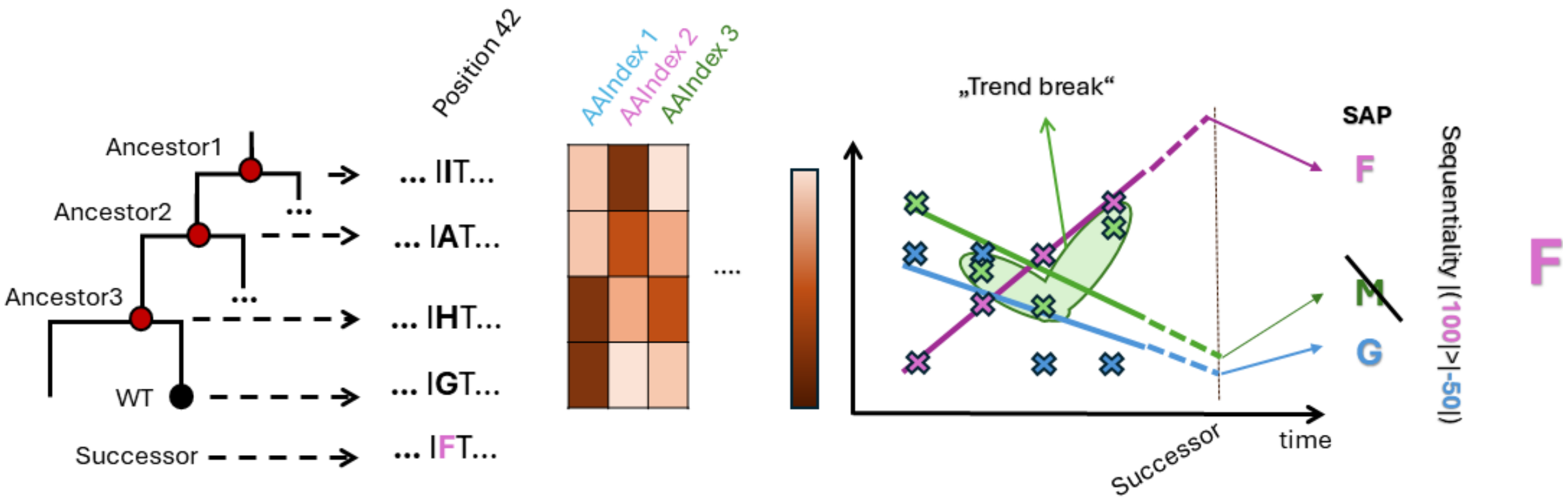

Figure S1: Illustration of amino acid prediction at position 42 in the successor sequence prediction pipeline. For simplicity, three AAindices with different colors are highlighted, with their corresponding index values shown in the orange scale. **(Left)** The evolutionary trajectory at position 42 is reconstructed by tracing the main path from the root through ancestral nodes (Ancestor1→Ancestor2→Ancestor3) down to the wild-type (WT) sequence. The amino acid at each node is read from the multiple sequence alignment, here changing from I→A→H→G across the trajectory, with F predicted as the successor (see next panels). **(Center)** For each node in the trajectory, the amino acid at position 42 is translated into a numerical feature value using each of the three selected AAindices (shown as colored columns in the heatmap). Darker shading indicates a lower index value. Each column thus represents how one physicochemical property changes along the evolutionary path from root to WT. **(Right, plot)** For each AAindex, a linear regression is fit to the distance-weighted feature values (crosses) across the trajectory nodes. The regression line is extrapolated one step beyond the WT to predict the successor feature value (dashed vertical line). The green line (AAindex 3) shows a clear downward trend, but its penultimate transition deviates from that overall direction, flagged as a **trend break**, so its prediction is discarded. The magenta (AAindex 2) and blue (AAindex 1) lines have no trend break at the penultimate position and yield extrapolated values whose nearest amino acid match is used as the prediction. This is a case of a site agreeing prediction **(SAP)**, as predictions do not agree on substitution. Please note that in the original pipeline, the third approved prediction would have led this position to be considered SAP, but we do not show it here for figure clarity. **(Right, panel)** Predictions from each AAindex are ranked by a composite score incorporating sequentiality, frequency among predictions, and extrapolation error (the difference between the extrapolated feature value and the true AAindex value of the predicted amino acid). In this example, each index predicts a different amino acid substitution (frequency = 1 for all), and the extrapolation error is assumed to be zero for all, so sequentiality alone determines the ranking. The magenta index achieves a higher sequentiality score because each value along its trajectory is strictly higher than the previous one, reflecting a perfectly consistent trend, whereas the blue index shows a less consistent trend, with inconsistent increases/decreases at each step along the trajectory. The magenta index is therefore selected as the final SAP prediction (**F**).

Successor sequence predictor - single tree pipeline

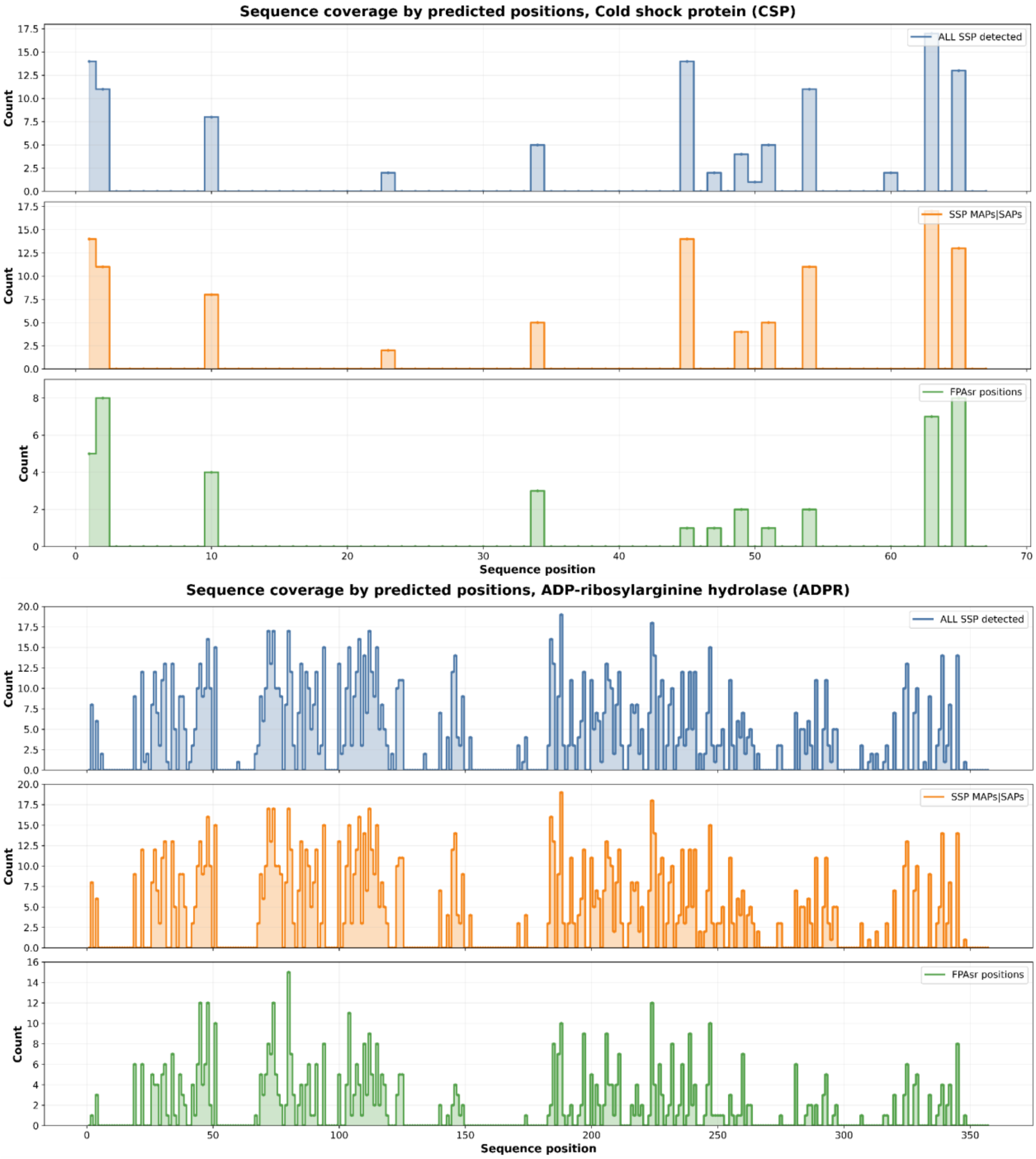

Figure S2: Sensitivity analysis of the modified single-tree SSP workflow implemented in FireProtASR 2.0. Successor predictions were generated for the query proteins from the Cold Shock Protein dataset using 131 phylogenetic trees (top) and the ADP-ribosylarginine hydrolase dataset using 87 phylogenetic trees (bottom). Blue indicates all successor mutations predicted by the original multiple-tree consensus SSP (mSSP), orange denotes the final SAP/MAP predictions selected by mSSP, and green represents the distribution of predictions obtained from individual single-tree SSP (sSSP) runs. The number of predictions generated by individual sSSP runs varies between 0 and 20 for the ADP-ribosylarginine hydrolase dataset and between 0 and 3 for the Cold Shock Protein dataset, reflecting the dependence of the prediction outcome on the selected phylogenetic tree. Although the single-tree workflow yields fewer predictions than the original mSSP approach, the resulting predictions represent a conservative subset of the original prediction space while preserving its diversity.

References

1. Rosinska M, Svobodova L, Borko S, Lacko D, Planas-Iglesias J, Marques SM, Kabourek P, Liu B, Pailozian K, Damborsky J, Mazurenko S, Bednar D. EnzymeMiner 2.0: advancing automated enzyme discovery with expansive sequence mining and smart property analysis. Nucleic Acids Research. 2026 May 11:gkag424, https://doi.org/10.1093/nar/gkag424
2. Khan RT, Kohout P, Musil M, Rosinska M, Damborsky J, Mazurenko S, Bednar D. Anticipating protein evolution with successor sequence predictor. Journal of Cheminformatics. 2025 Mar 21;17(1):34.. https://doi.org/10.1186/s13321-025-00971-z
3. Kohout P, Vasina M, Majerova M, Novakova V, Damborsky J, Bednar D, Marek M, Prokop Z, Mazurenko S. Engineering dehalogenase enzymes using variational autoencoder-generated latent spaces and microfluidics. JACS Au. 2025 Feb 13;5(2):838-50. https://doi.org/10.1021/jacsau.4c01101
